# Tactile Biofeedback That Targets Stance Time Acutely Modulates Propulsion Mechanics in Healthy Gait

**DOI:** 10.21203/rs.3.rs-10045980/v1

**Published:** 2026-07-06

**Authors:** Christopher Engsberg, Nathaniel Hunt, Philippe Malcolm, Mukul Mukherjee

**Affiliations:** University of Nebraska at Omaha; University of Nebraska at Omaha; University of Nebraska at Omaha; University of Nebraska at Omaha

**Keywords:** Stimulation, Biofeedback, Temporal, Sensory, Coordination, Walking

## Abstract

Biofeedback is effective in modifying and improving targeted gait parameters in both healthy individuals and those with chronic stroke. However, prior studies have largely focused on the variable targeted by biofeedback and rarely report changes in other gait features. Given the interdependent nature of gait, alterations in one parameter may induce changes in other features. Thus, we aimed to determine whether tactile biofeedback to the plantar surfaces instructing stance time increases would increase propulsive gait features, such as trailing limb angles (TLA), peak propulsive forces, and propulsive impulses. 8 healthy individuals walked with tactile stimulation instructing varying levels of stance time increase targets (small, medium, and large) to one limb at a time. These stance time targets were calculated using symmetry ratios ranging from 0.775–1.225. This feedback instructed subjects to delay their push off, and thus their toe off, after a heel strike. During the large stance time increase feedback target, subjects increased their stance time (0.23 ± 0.02 seconds, p < 0.001), and consequently their TLA (8.1 ± 0.8°, p = 0.001), peak propulsion (8.1 ± 1.1%BW, p = 0.002), and their propulsive impulse (2.9 ± 0.3%BW·s, p = 0.002) compared to no feedback. Stance time changes were significantly correlated to TLA (R^2^ = 0.80, p < 0.001), and propulsive impulse (R^2^ = 0.81, p < 0.001), but to a lesser degree for peak propulsive force (R^2^ = 0.72, p < 0.001) when accounting for subject variances. These results provide evidence for a biofeedback tool that influences propulsive features that only instructs stance times. This may demonstrate the future application of a cheaper alternative for stroke survivors to improve the propulsion of the paretic limb compared to external assistances such as exoskeletons or ground reaction force biofeedback. Such a device could be an effective tool in post-stroke gait rehabilitation, for targeting improvements in the temporal and propulsive function of the paretic limb at the same time.

## Introduction

Some of the major problems that impact the gait of stroke survivors are their temporal and propulsive asymmetries (Awad, Lewek, et al., 2020; McCain et al., 2019; Patterson et al., 2008). These deficits contribute to increased metabolic costs (Cruz et al., 2009; Farris et al., 2015; Olney & Richards, 1996), reduced walking speed (Awad, Lewek, et al., 2020; Lauziere et al., 2014), and may contribute to reduced balance control (Hendrickson et al., 2014; Stimpson et al., 2019; Woollacott & Tang, 1997). Temporal improvements in stroke survivors are of particular importance, due to an apparent resistance to change (Kahn & Hornby, 2009; Reisman et al., 2007). The may be due to asymmetrical weight transfer between the paretic and nonparetic limbs, as effective weight bearing has been suggested to be necessary for a sufficient push off (Kim & Eng, 2003; Lauziere et al., 2014). Given that stance time is strongly related to the weight bearing on a limb (Kim & Eng, 2003), improving stance time on the paretic side may lead to concurrent improvements in propulsion. This is supported by simultaneous increases in both stance time and propulsion with biofeedback in healthy subjects (Schenck & Kesar, 2017) and amputees (Brandt et al., 2019), although their direct relationship has not been analyzed. If stance time improvements increase propulsion, this would support simultaneous training of temporal and propulsive deficits in stroke survivors.

Biofeedback has been effective for improving the stance time and, in separate studies, propulsive features in individuals with chronic stroke (Drużbicki et al., 2016; Genthe et al., 2018; Lewek et al., 2012; Santucci et al., 2023). However, most of these biofeedback techniques were provided in the context of the target variable’s symmetry (Drużbicki et al., 2016; Kettlety et al., 2025; Lewek et al., 2012). Following this type of feedback may be difficult, and lead to unwanted changes in the nonparetic limb (Padmanabhan et al., 2020). For example, when individuals with chronic stroke attempt to reduce step length asymmetry, they exhibit reduced balance (Park et al., 2021), which may be attributed to increased compensatory movements (Cruz et al., 2009). Instructing only the paretic limb may reduce these compensatory movements and promote a more desirable gait pattern. For these reasons, we aimed to determine how instructing one limb at a time affects stance time and, consequently, propulsion in healthy individuals, with the goal of future application in those with chronic stroke.

Therefore, the aims of the current study were to determine (1) if healthy individuals could accurately alter their stance times according to tactile biofeedback applied to the plantar surfaces of the feet, and (2) if these stance time changes lead to significant increases in TLA, propulsive force, and propulsive impulse. We hypothesized that tactile feedback could have specificity for stance times of a limb, such that small, medium, and large instructed increases would be significantly different from one another. Additionally, to determine whether the steps following that feedback also have significantly larger TLA, peak propulsion, and propulsive impulse than steps without feedback. Lastly, we hypothesized that stance time would be significantly positively related to TLA, peak propulsive force, and propulsive impulse.

## Methods

### Subjects

Eight healthy adults (27.6 ± 3.8 years, 5 males) participated in the study (Table 1), with exclusion criteria including any dysfunction, such as physical impairments, neurological disease, or other abnormalities that may affect walking. All subjects provided informed consent, and the internal review board of the University of Nebraska Medical Center approved the study.

### Equipment

**Figure 1:**
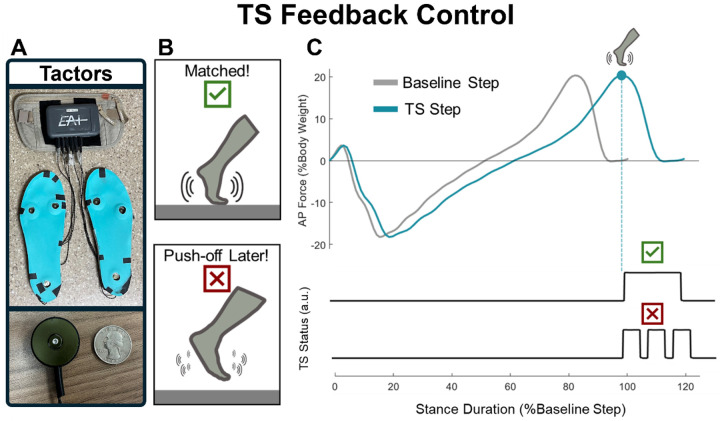
Plantar tactile stimulation (TS) instructed increases to stance time. (A) Subjects were fitted with custom-sized insoles containing tactors. Subjects were instructed to push off the ground at the onset of the vibration and were shown diagram (B) to aid understanding. If they reached the instructed target, they were provided with ‘success’ feedback (a constant vibration); if they did not reach the target, they were provided with ‘failure’ feedback (a pulsing vibration). (C) Example of feedback instructing a 20% increase in stance time. The onset of vibration was 20% later than their estimated peak propulsion time after a heel-strike. The baseline step (grey) would receive the ‘failure’ feedback, while the TS step (blue) would receive the ‘success’ feedback.

Reflective markers were attached to the subjects according to a modified lower limb Helen Hays marker set to obtain lower limb and pelvic kinematics. Marker trajectories were recorded at 100 Hz using a 16-camera motion capture system (Vicon, Oxford, UK). Subjects walked on a force plate instrumented split-belt treadmill (Bertec Version 2.0 2013, Columbus, OH) with ground reaction forces (GRF) collected at 1000 Hz. Tactile stimulation was controlled via a custom real-time MATLAB controller (Mathworks, Natick, MA), described previously (Engsberg et al., 2024). This controller received marker and force data at 100 Hz via the Nexus DataStreamSDK to identify gait events (heel strikes and toe-offs) and peak propulsive force timing from the anteroposterior (AP) GRFs. The controller sent activation signals to a waist-mounted amplifier box powering six vibrating tactors (Engineering Acoustics Inc., Casselberry, FL), with three embedded in each custom insole (Fig. 1A) worn inside the provided shoes (Free running shoes, Nike, Beaverton, OR). Each tactor was 0.78 cm thick, 3.05 cm in diameter, 17.0 g in mass, and were set to vibrate at 250 Hz and an amplitude of 23.5 dB (0.2 mm).

### Tactile Biofeedback Instruction

The tactile stimulation (TS) used a real-time controller to determine the average stance times and the estimated time of peak propulsion from each subject. Subjects were instructed to push off the ground when they felt a vibration on their foot. Thus, vibrations felt later in stance (or during swing) prompted increases to that limb’s stance time. To improve clarity and motivation, subjects received a ‘success’ or ‘failure’ vibration depending on whether the previous step met the stance time target. If the limb’s previous stance time was equal to or greater than the target, the vibration was a constant pulse for 300ms. If the stance time was less than the target, they received pulsing feedback comprised of quick 40ms vibrations separated by 20ms of no vibrations for 300ms (Fig. 1C). Only one limb was provided vibrations at a time.

Real-time heel strikes and toe-offs were used to calculate the subjects’ stance times. A heel strike was defined as when the AP velocity of the heel marker crossed zero, moving from positive (forward) to negative (backward), and vice versa for toe-offs (Engsberg et al., 2024; Zeni et al., 2008). Stance time was defined as the time from a heel-strike to the subsequent ipsilateral toe-off. Target stance time calculations were performed using the most recent 10 steps during times of no tactile stimulation (no TS) from the left and right limbs (*StcT*_*L*_ and *StcT*_*R*_). The timing of the peak propulsive force between the two limbs was also calculated from the real-time force plate data for those 10 steps. This was converted into a percentage of stance (*PeakTime*) for calculating the target stance time (*StcT*_*Lnew*_ or *StcT*_*Rnew*_) and the timing of stimulation onset (*TS*_*Oset*_). These targets were calculated using symmetry ratios (*Ratio*_*Cond*_) to obtain either small, medium, or large increases in stance time (see Procedure). It is important to note that ratios that were greater than 1.0 were applied to the left foot (Eq. 1), and ratios less than 1.0 were for the right foot (Eq. 2) the target stance times were used in Eq. 3. Thus, stance times were only instructed to increase for one foot at a time. A ratio of 1.0 (BaselineTS) was applied to the left foot for the first experimental trial and to the right foot on the second trial.


Equation 1: $${\backslash \text{varvec}S\backslash \text{varvec}t\backslash \text{varvec}c\backslash \text{varvec}T}_{\backslash \text{varvec}L\backslash \text{varvec}n\backslash \text{varvec}e\backslash \text{varvec}w}={\backslash \text{varvec}S\backslash \text{varvec}t\backslash \text{varvec}c\backslash \text{varvec}T}_{\backslash \text{varvec}R}•\backslash \text{varvec}R\backslash \text{varvec}a\backslash \text{varve}$$

Equation 2: $${\backslash \text{varvec}S\backslash \text{varvec}t\backslash \text{varvec}c\backslash \text{varvec}T}_{\backslash \text{varvec}R\backslash \text{varvec}n\backslash \text{varvec}e\backslash \text{varvec}w}=\backslash \text{varvec}S\backslash \text{v}$$

Equation 3: $${\backslash \text{varvec}T\backslash \text{varvec}S}_{\backslash \text{varvec}O\backslash \text{varvec}n\backslash \text{varvec}s\backslash \text{varvec}e\backslash \text{varvec}t}=\backslash \text{varvec}S\backslash \text{varvec}t\backslash \text{varv}$$

### Procedure

Subjects first completed a 5-minute treadmill familiarization at 1.0 m/s, which was the walking speed for all trials. This speed was selected to minimize the confounding effects of walking speed and to ensure participants could modify their gait without constraint, allowing them to adjust their stepping pattern in response to the feedback. After completing a 3-minute baseline walking trial, subjects were provided with instructions on how to use the tactile biofeedback. They were told to synchronize their push-off from their stimulated limb with the onset of vibrations, such that a delayed vibration after a heel-strike would increase stance time. The vibration type also indicated their performance, where the ‘success’ constant vibration signaled reaching the target stance time, and the ‘failure’ pulsing vibration indicated not reaching it (see Tactile Biofeedback Instruction). Subjects then completed a 5-min biofeedback familiarization trial with varying stance time targets and were encouraged to ask questions until confident in following the feedback.

Finally, two experimental trials (8.75 minutes each) were performed, each consisting of seven 45-second TS periods, separated by 30 sec of no TS to allow for returning to a normal walking pattern (Schenck & Kesar, 2017). The TS periods had various stance time targets, with symmetry ratios incrementing by 0.075. For the left limb, ratios of 1.0, 1.075, 1.15, and 1.225; for the right limb, ratios of 1.0, 0.925, 0.85, and 0.775 were used. These corresponded to BaselineTS (1.0), LeftTS_Sml_ (1.075), LeftTS_Med_ (1.15), LeftTS_Lrg_ (1.225), and so on for RightTS conditions (Equations 1–3). This method of symmetry ratios was chosen to align with previous temporal biofeedback studies (Afzal et al., 2015; Drużbicki et al., 2016; Khoo et al., 2017; Michelini et al., 2022) and enable future applications to asymmetric stroke populations. The order of these conditions was randomized for each subject and trial. Overall, this method yielded graded stance time targets for both limbs.

### Data Analysis

Post-processing of data was performed in MATLAB and statistical analysis was performed in R (R- 4.5.1). Stance times were calculated using the marker position data from the heel and toe markers to determine the time between an ipsilateral heel-strike and toe-off (Engsberg et al., 2024; Zeni et al., 2008). Propulsion variables included TLA, peak propulsion, and propulsive impulse. TLA was the maximum sagittal plane angle between the center of mass (CoM) and ipsilateral MT5 marker during double support (Lewek & Sawicki, 2019), with CoM estimated from the pelvic markers (Lewek et al., 2018). Propulsive forces were normalized to body weight (BW). Peak propulsion was the maximum AP GRF during stance, and propulsive impulse was the time integrated positive AP GRF for each limb (Lewek et al., 2018).

All stance times for each condition were used in a linear mixed effects model to determine whether TS feedback resulted in significant increases compared with no TS and to test specificity across conditions for hypothesis 1. Fixed effects included the interaction between feedback condition and limb (left/right), with subject as a random factor. Models were fit using the “lme4” package in R, with fixed effects tested via Type III ANOVAs. Effect sizes (partial η ^2^) were computed using the “effectsize” function, and estimated marginal means with Tukey-adjusted pairwise comparisons were obtained. Significance was set at α = 0.05. Propulsive outcomes were analyzed only for the limb receiving feedback (limb with the targeted stance time provided) and for steps during the no TS condition. This subset of data was chosen to remove the effect of side (left or right limb). A similar linear mixed-effects model was used with condition (no TS, BaselineTS, TS _Sml_, TS_Med_, and TS_Lrg_) as the only fixed effect for hypothesis 2. All other analysis steps matched the stance time analysis.

Relationships between stance time and propulsive features for hypothesis 3 were assessed using Spearman correlations and linear mixed effects models, consistent with prior work (M. Legrand et al., 2025; M. L. Legrand et al., 2024), and to account for subject effects. Normality was evaluated using Q-Q and density plots for the linear models. Spearman correlations were computed with the “cor.test” function, and coefficients were classified as fair (|r| = 0.3–0.5), moderate (0.6–0.7), or very strong (0.8–0.9) (Akoglu, 2018; Chan, n.d.).

## RESULTS

### Stance Times During TS Feedback

**Figure 2:**
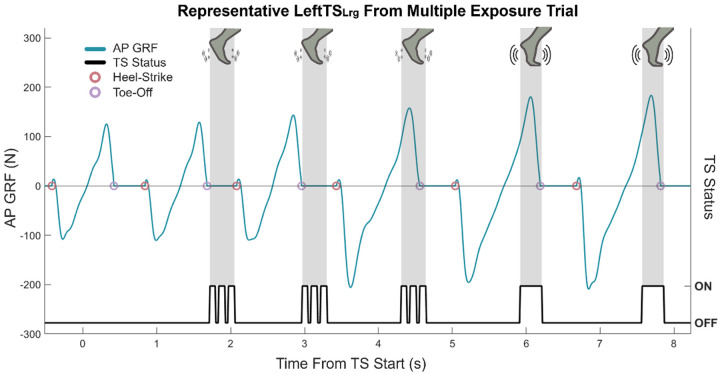
Representative subject altering their gait to match the LeftTS_Lrg_ tactile feedback. Zero on the x-axis marks the start of the LeftTS_Lrg_ condition. The left y-axis is the left limb’s propulsive GRF (blue line), while the right y-axis shows the stimulation status (black line). Success feedback is a constant vibration, while failure feedback is a pulsing vibration. Failure feedback instructed subjects to increase their stance time but was responsive to the previous step. Vibration duration is shown in grey shading. The time between heel-strikes (orange circles) and toe-offs (purple circles) increased until success feedback was provided.

**Figure 3:**
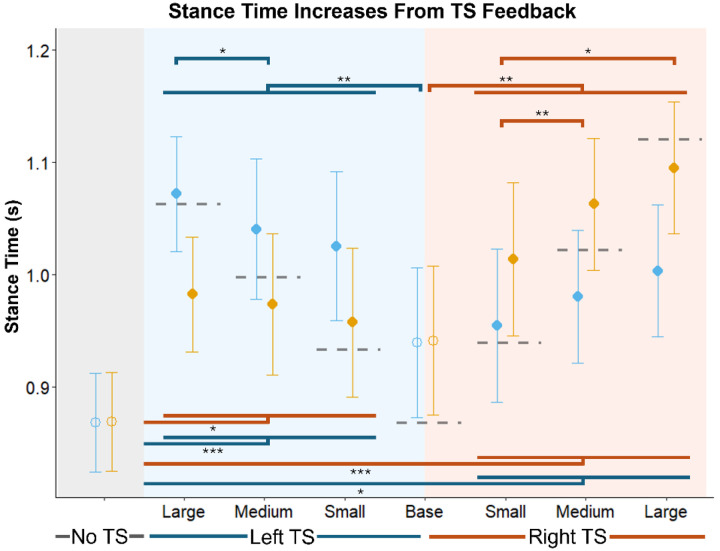
Subjects adjusted their stance times in response to TS feedback. Linear mixed effects estimated marginal means for left (blue dots) and right (orange dots) limbs across the stimulation conditions (n = 8). The limb receiving stimulation for each condition is indicated by background shading: no stimulation (grey), left foot stimulation (blue), right foot stimulation (orange). The grey dashed lines denote average stance time targets for subjects. Significant differences between conditions are denoted with brackets for the left limb (blue brackets) and right limb (orange brackets). Filled dots denote a significant difference between left and right values for that instructed condition. Error bars are 95% confidence intervals. (*p<0.05, **p<0.01, ***p<0.001).

Subjects took about 60 steps for each tactile instructed condition, an example of following the LeftTS _Lrg_ condition is shown in Fig. 2. The linear mixed effect ANOVA showed a significant main effect of condition (F = 70.76, partial η2 = 0.98, p < 0.001), no main effect for side (F = 1.14, p = 0.28), and a significant interaction effect (F = 327.08, partial η^2^ = 0.16, p < 0.001). Post-hoc pairwise comparisons showed that all instructed conditions, except BaselineTS, significantly increased stance time compared to no TS (RightTS_Lrg_ = + 0.23 ± 0.02 s, RightTS_Med_ = + 0.19 ± 0.02 s, RightTS_Sml_ = + 0.15 ± 0.02 s.; LeftTS_Lrg_ = + 0.20 ± 0.02 s, LeftTS_Med_ = + 0.17 ± 0.02 s, LeftTS_Sml_ = + 0.16 ± 0.02 s). However, differences between incremental levels were limited. Condition specificity was observed primarily between BaselineTS and all other conditions for the stimulated limb, as well as between RightTS_Sml_ vs. RightTS_Med_ and RightTS_Lrg_, and LeftTS_Med_ vs. LefetTS_Lrg_ (Fig. 3). Between-limb differences were absent only in the no TS and BaselineTS conditions (Fig. 3). The uninstructed limb only significantly increased stance time for the small, medium, and large feedback targets.

### Propulsion During Stance Time Feedback

**Figure 4:**
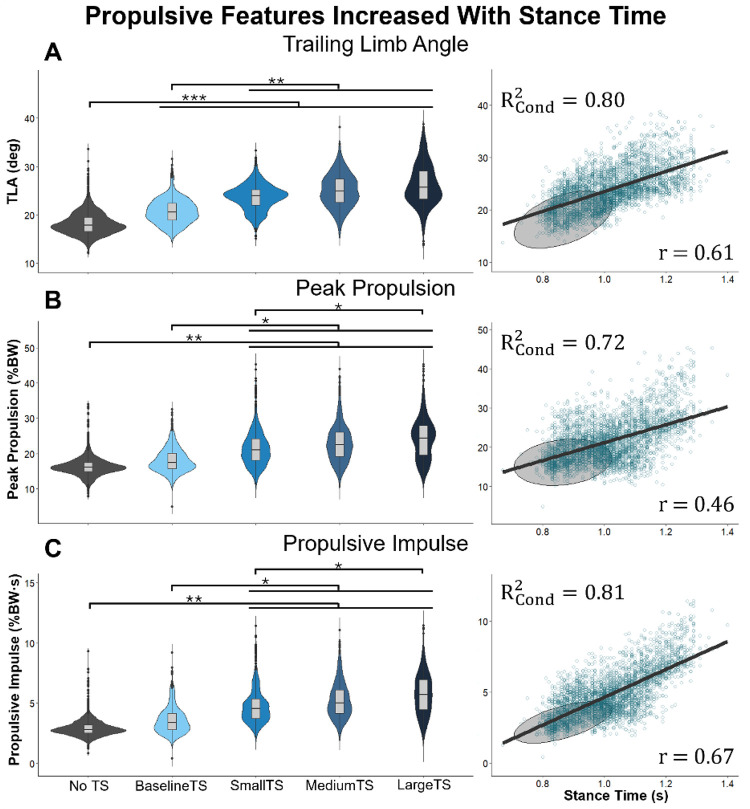
Propulsive features of gait significantly increased during stance time with tactile feedback. Violin plots of linear mixed effects model results from (A) TLA, (B) peak propulsive force, and (C) propulsive impulse (n = 8). TS conditions resulted in significant increases in all three features; however, additional specificity of the feedback condition was present for propulsion peaks and impulses. Linear regressions of the same propulsive features (n = 8) are shown to the right of violin plots with their respective conditional R^2^ fit and Spearman correlation values. Each dot represents an individual step when it received tactile feedback; the grey ellipse is the 95% confidence interval for the No TS condition for reference (not included in the regression fit).

The models determined a significant effect of TS condition for each propulsive feature (TLA: F = 16.86, p = 0.001, η^2^ = 0.90; Peak Propulsion: F = 12.54, p = 0.003, η^2^ = 0.88; Propulsive Impulse: F = 10.92, p = 0.004, η^2^ = 0.86). For TLA, all stimulation conditions were significantly greater than no TS, but only BaselineTS differed from the other stimulation conditions; no further specificity was found. Peak propulsion and propulsive impulse showed similar trends: BaselineTS did not differ from no TS but differed from all other conditions, with additional specificity between the small and large TS targets (Fig. 4A–C).

For correlation analysis, a subset of the total sample of steps was chosen, including only steps from the stimulated limb (LeftTS: left steps, RightTS: right steps) and the absence of no TS steps. This was done so the correlations reflected the variation present in the limb that received the feedback, and to reduce the overrepresentation of no TS steps (see Supplemental Figure S2 for full data correlations). Normality tests from the data set are provided in the Supplemental Material (Figure S1). All Spearman correlations were significant. Stance time had a moderate correlation with propulsive impulse (r = 0.67, p < 0.001) and TLA (r = 0.61, p < 0.001), and a fair correlation with peak propulsive force (r = 0.46, p < 0.001) (Fig. 4A–C). Linear mixed effects models accounting for subject variability showed similar variance explanation for TLA (R_Mar_^2^ = 0.51, R_Cond_^2^ = 0.80, p < 0.001) and propulsive impulse (R_Mar_^2^ = 0.60, R_Cond_^2^ = 0.81, p < 0.001), but were worse for peak propulsive force (R_Mar_^2^ = 0.41, R_Cond_^2^ = 0.72, p < 0.001).

## Discussion

This study aimed to determine whether temporal tactile feedback could effectively alter stance times and, in turn, the propulsive features of healthy subjects. These results partially support the ability to specifically alter stance times, with various instructed targets resulting in significantly different stance times for the subjects. The biofeedback only caused different stance times between the large and medium conditions for the left limb, and between the small and the medium and large conditions for the right limb. However, we found supporting evidence for our second hypothesis, as these same feedback targets resulted in significant increases in propulsive features, such as TLA, peak propulsive force, and propulsive impulse. Finally, we demonstrate a significant relationship between stance time and these propulsive features, supporting our final hypothesis.

### Specificity Stance Time Instruction

Stance time instruction was provided to only one limb at a time; however, both limbs tended to increase their stance times during the feedback. This may be from the tendency for individuals to walk symmetrically (Mukherjee et al., 2015; Reisman et al., 2007; Sado et al., 2022). Even so, the limb being instructed consistently had a longer stance time than the uninstructed limb, and after the initial increase in the uninstructed limb, it did not increase further (Fig. 3). Specificity between levels of instructed stance times was present for both limbs on the margins between the RightTS_Med_ and RightTS_Sml_ conditions for the right limb (about 0.08 s difference on average) and LeftTS_Lrg_ and LeftTS_Med_ for the left limb (about 0.065 s difference on average). This demonstrates the biofeedback device’s ability to instruct a specific increase in stance time for a limb. Few biofeedback studies have specific stance time targets (Brandt et al., 2019; Brandt & Huang, 2019), rather than symmetry value targets (Afzal et al., 2015; Georgiou et al., 2020; Michelini et al., 2022). Although target values were not reported, amputees could increase stance time by a margin of ~ 0.06 s on average with visual feedback (Brandt & Huang, 2019). Here, we demonstrate similar control using tactile feedback. Finally, the RightTS_Lrg_ target appeared too large for some subjects (Fig. 3), requiring ~ 0.25 s increases, whereas LeftTS_Lrg_ (~ 0.20 s increase) was generally achievable. This suggests an upper limit to stance time increases during constant-speed treadmill walking. Accuracy analysis is provided in the **Supplemental Material**.

### Stance Time Instruction Results in Propulsion Increases

This tactile feedback provided only temporal instruction; nonetheless, significant increases in key propulsive variables were observed (Fig. 4). The connection between stance time and peak propulsion has been shown previously in healthy subjects using visual biofeedback in the context of symmetry, with a reported Spearman correlation of 0.56 between peak propulsive force and stance time asymmetries (M. Legrand et al., 2025). However, in the current study, we report the step-by-step values for individual limbs. So, it is interesting that both the coordination between the limbs and the direct force applied to the ground are related to stance time. Here, we expand on these findings by showing the relationship between stance time and multiple propulsive features. Interestingly, only TLA was found to significantly increase during the BaselineTS feedback condition (Fig. 4A). This could be from the feedback providing instruction at terminal stance, leading to subjects exaggerating their trailing limb kinematics while not significantly impacting the forces applied to the ground, however the found Spearman correlation between TLA and peak propulsion (r = 0.77, p < 0.001) is within a similar range to what has been reported for stroke survivors (r = 0.70) (Lewek & Sawicki, 2019). Further investigation is required to determine how healthy subjects may achieve greater control of TLA without affecting GRF propulsion.

Stance time was related to all tested propulsive variables, suggesting a potential mechanism linking temporal modulation to propulsion during treadmill walking. Increasing stance time at a constant speed increases limb excursion (Hoogkamer et al., 2014), placing the foot further behind the CoM at terminal stance, thereby, increasing TLA. TLA increases are strongly associated with larger propulsive forces (Lewek & Sawicki, 2019). Increased propulsion over a longer duration due to the larger stance time, would lead to greater impulse. This cumulative effect may explain why the strongest relationship observed was between stance time and propulsive impulse (Fig. 4C). It would be interesting to determine whether the effect of stance times on propulsion could be directly modeled to support this proposed mechanism. Notably, this relationship does not appear driven by feedback timing at push off, as the opposite limb during stimulation (e.g., right stance time during LeftTS_Lrg_) showed a similar association (r = 0.62; **Supplemental Figure S3**). This suggests the relationship between stance time and propulsion stems from the stance time instruction rather than the timing of the tactile feedback.

Overall, our biofeedback targeting increased stance time resulted in significant modulation of propulsion variables. This demonstrates a method for influencing a subject’s propulsive forces using simple temporal feedback. Such a feedback mechanism could be beneficial for individuals who have reduced propulsive and spatiotemporal capabilities, such as chronic stroke survivors.

### Use in Stroke Survivors

This study demonstrates an effective method to increase stance times and propulsion in healthy subjects. Although increasing stance times outside preferred ranges raises energetic costs (Collins & Jackson, 2013), this approach may benefit chronic stroke survivors. The reduced paretic propulsion in stroke survivors (Awad, Lewek, et al., 2020; McCain et al., 2019) likely contributes to the increased metabolic cost of hemiparetic gait (Farris et al., 2015; Olney & Richards, 1996), suggesting that improving propulsive symmetry may reduce this cost. Exoskeletons can achieve this (Awad et al., 2017; Awad, Kudzia, et al., 2020; McCain et al., 2019), however, this is from external assistance, not from the stroke survivors’ own walking mechanics. In contrast, our tactile device may increase paretic propulsion by leveraging the found stance time and propulsion relationship. At the same time, this could improve the temporal aspects of the paretic limb – an important rehabilitation goal (Hendrickson et al., 2014; Patterson et al., 2010). Previous temporal (Khoo et al., 2017; Lewek et al., 2012; Skvortsov et al., 2021) and propulsive (Genthe et al., 2018; Kettlety et al., 2025; Santucci et al., 2023) biofeedback studies are effective independently in chronic stroke survivors. Here, we propose that stance time feedback could be a unified strategy to improve both the temporal and propulsive capabilities of the paretic limb during walking.

It is important to note that these results were obtained from young, healthy individuals walking on a treadmill at a fixed speed. Prior biofeedback studies have demonstrated that temporal gait features can be modified in individuals with stroke (Afzal et al., 2015; Lewek et al., 2012), suggesting that the ability to use the current tactile device may not be a barrier to eliciting similar gait adaptations in hemiparetic populations. However, overground walking introduces additional velocity and walking strategies compared to treadmill walking, which may alter the relationship between stance time and propulsion. Therefore, future work will extend this approach using a portable device to evaluate the effects of stance time modulation in individuals with stroke during overground walking. In an overground setting, a device that relies solely on stance time to guide increases in propulsive features could provide a low-cost alternative to portable exoskeletons or biofeedback of ground reaction forces. If individuals with stroke demonstrate responses similar to those observed in healthy participants, this would support its potential as an accessible tool for improving temporal and propulsive gait function in chronic stroke.

### Limitations

The sample size in the current study is small; however, a post-hoc power analysis of our data indicates strong power (> 0.9). Performing a stepwise analysis helped improve our power, given our small sample size. We did not directly report the accuracy of subjects in following the tactile feedback; however, these values are reported in the **Supplemental Material**.

## Conclusion

This study demonstrates that providing tactile biofeedback to the soles of the feet is an effective method for selectively increasing stance times in healthy subjects. This stance time modulation also resulted in significant increases and strong correlations to key propulsive variables – TLA, peak propulsive force, and propulsive impulse – at the same time. This supports a simple feedback strategy for manipulating propulsion via stance time instructions. If these responses persist in neurologically impaired subjects, such as stroke survivors, it will improve the temporal and propulsive capabilities of the paretic limb while walking.

## Supplementary Material

Supplementary Files

This is a list of supplementary files associated with this preprint. Click to download.

- HapticbiofeedbackofstancetimesSUPP.docx

## Figures and Tables

**Table 1:** Subject demographics.

Subject	Sex	Age	Height (cm)	Weight (kg)	Leg Dominance	Leg Length (cm)	Shoe Size (cm)
1	M	26	176	74.4	R	98.5	27.9
2	F	22	156	49.9	R	75.4	23.8
3	M	27	190	82.5	R	103.5	28.6
4	M	36	173	85.7	R	91.5	26.0
5	M	28	180	88.4	R	93.5	28.3
6	M	30	180	93.0	R	93.0	27.9
7	F	25	163	51.7	R	84.0	24.1
8	F	27	179	63.0	R	92.5	27.3
**Average**		**27**	**175**	**73.6**		**91.5**	**26.7**
Standard Deviation		3.8	10.0	15.7		8.0	1.8

## Data Availability

All data supporting the findings of this study are available within the paper and its Supplementary Information

## References

[1] Afzal M. R., Oh M.-K., Lee C.-H., Park Y. S., & Yoon J. (2015). A Portable Gait Asymmetry Rehabilitation System for Individuals with Stroke Using a Vibrotactile Feedback. BioMed Research International, 2015, 1–16. 10.1155/2015/375638 PMC4486481 26161398

[2] Akoglu H. (2018). User’s guide to correlation coefficients. Turkish Journal of Emergency Medicine, 18(3), 91–93. 10.1016/j.tjem.2018.08.001 30191186 PMC6107969

[3] Awad L. N., Bae J., O’Donnell K., De Rossi S. M. M., Hendron K., Sloot L. H., Kudzia P., Allen S., Holt K. G., Ellis T. D., & Walsh C. J. (2017). A soft robotic exosuit improves walking in patients after stroke. Science Translational Medicine, 9(400), eaai9084. 10.1126/scitranslmed.aai9084 28747517

[4] Awad L. N., Kudzia P., Revi D. A., Ellis T. D., & Walsh C. J. (2020). Walking Faster and Farther With a Soft Robotic Exosuit: Implications for Post-Stroke Gait Assistance and Rehabilitation. IEEE Open Journal of Engineering in Medicine and Biology, 1, 108–115. 10.1109/OJEMB.2020.2984429 33748765 PMC7971412

[5] Awad L. N., Lewek M. D., Kesar T. M., Franz J. R., & Bowden M. G. (2020). These legs were made for propulsion: Advancing the diagnosis and treatment of post-stroke propulsion deficits. Journal of NeuroEngineering and Rehabilitation, 17(1), 139. 10.1186/s12984-020-00747-6 33087137 PMC7579929

[6] Brandt A., & Huang H. H. (2019). Effects of extended stance time on a powered knee prosthesis and gait symmetry on the lateral control of balance during walking in individuals with unilateral amputation. Journal of NeuroEngineering and Rehabilitation, 16(1), 151. 10.1186/s12984-019-0625-6 31783759 PMC6883569

[7] Brandt A., Riddick W., Stallrich J., Lewek M., & Huang H. H. (2019). Effects of extended powered knee prosthesis stance time via visual feedback on gait symmetry of individuals with unilateral amputation: A preliminary study. Journal of NeuroEngineering and Rehabilitation, 16(1), 112. 10.1186/s12984-019-0583-z 31511010 PMC6737689

[8] Chan Y. H. (n.d.). Biostatistics 104: Correlational Analysis (Vol. 44, pp. 614–619). Singap. Med. J. 14770254

[9] Collins S. H., & Jackson R. W. (2013). Inducing self-selected human engagement in robotic locomotion training. 2013 IEEE 13th International Conference on Rehabilitation Robotics (ICORR), 1–6. 10.1109/ICORR.2013.6650488 24187305

[10] Cruz T. H., Lewek M. D., & Dhaher Y. Y. (2009). Biomechanical impairments and gait adaptations post-stroke: Multi-factorial associations. Journal of Biomechanics, 42(11), 1673–1677. 10.1016/j.jbiomech.2009.04.015 19457488 PMC3641760

[11] Drużbicki M., Guzik A., Przysada G., Kwolek A., Brzozowska-Magoń A., & Sobolewski M. (2016). Changes in Gait Symmetry After Training on a Treadmill with Biofeedback in Chronic Stroke Patients: A 6-Month Follow-Up From a Randomized Controlled Trial. Medical Science Monitor, 22, 4859–4868. 10.12659/MSM.898420 27941712 PMC5170889

[12] Engsberg C. P., Hunt N., & Mukherjee M. (2024). Gait Kinematic Dependent Plantar Stimulation. 2024 46th Annual International Conference of the IEEE Engineering in Medicine and Biology Society (EMBC), 1–4. 10.1109/EMBC53108.2024.10782134 PMC12035878 40039559

[13] Farris D. J., Hampton A., Lewek M. D., & Sawicki G. S. (2015). Revisiting the mechanics and energetics of walking in individuals with chronic hemiparesis following stroke: From individual limbs to lower limb joints. ring and Rehabilitation, 12(1), 24. 10.1186/s12984-015-0012-x PMC4357211 25889030

[14] Genthe K., Schenck C., Eicholtz S., Zajac-Cox L., Wolf S., & Kesar T. M. (2018). Effects of real-time gait biofeedback on paretic propulsion and gait biomechanics in individuals post-stroke. Topics in Stroke Rehabilitation, 25(3), 186–193. 10.1080/10749357.2018.1436384 29457532 PMC5901660

[15] Georgiou T., Holland S., & Van Der Linden J. (2020). Rhythmic Haptic Cueing for Gait Rehabilitation of People With Hemiparesis: Quantitative Gait Study. JMIR Biomedical Engineering, 5(1), e18649. 10.2196/18649

[16] Hendrickson J., Patterson K. K., Inness E. L., McIlroy W. E., & Mansfield A. (2014). Relationship between asymmetry of quiet standing balance control and walking post-stroke. Gait & Posture, 39(1), 177–181. 10.1016/j.gaitpost.2013.06.022 23877032

[17] Hoogkamer W., Bruijn S. M., & Duysens J. (2014). Gait Parameters Affecting the Perception Threshold of Locomotor Symmetry: Comment on Lauzière, *et al*. (2014). Perceptual and Motor Skills, 119(2), 474–477. 10.2466/25.15.PMS.119c22z8 25244554

[18] Kahn J., H., & Hornby T. G. (2009). Rapid and long-term adaptations in gait symmetry following unilateral step training in people with hemiparesis. Physical Therapy & Rehabilitation Journal, 89(5), 474–483. 10.2522/ptj.20080237 19282361 PMC2676432

[19] Kettlety S. A., Finley J. M., & Leech K. A. (2025). Within-session propulsion asymmetry changes have a limited effect on gait asymmetry post-stroke. Journal of NeuroEngineering and Rehabilitation, 22(1), 9. 10.1186/s12984-025-01553-8 39844188 PMC11756213

[20] Khoo I.-H., Marayong P., Krishnan V., Balagtas M., Rojas O., & Leyba K. (2017). Real-time biofeedback device for gait rehabilitation of post-stroke patients. Biomedical Engineering Letters, 7(4), 287–298. 10.1007/s13534-017-0036-1 30603178 PMC6208514

[21] Kim C. M., & Eng J. J. (2003). Symmetry in vertical ground reaction force is accompanied by symmetry in temporal but not distance variables of gait in persons with stroke. Gait & Posture, 18(1), 23–28. 10.1016/S0966-6362(02)00122-4 12855297

[22] Lauziere S., Betschart M., Aissaoui R., & Nadeau S. (2014). Understanding Spatial and Temporal Gait Asymmetries in Individuals Post Stroke. International Journal of Physical Medicine & Rehabilitation, 02(03). 10.4172/2329-9096.1000201

[23] Legrand M., Grenet F., Hochstrasser O., Luft A., Gassert R., Lambercy O., & Awai C. E. (2025). Real-time augmented feedback for gait training: Are gait responses affected by the choice of target parameter? Frontiers in Bioengineering and Biotechnology, 13, 1645390. 10.3389/fbioe.2025.1645390 40861862 PMC12371336

[24] Legrand M. L., Magrini C., Branscheidt M., Luft A., Gassert R., Lambercy O., & Easthope C. A. (2024). Augmented Feedback to Influence Gait Symmetry: A Feasibility Study to Quantify Effects on the Global Gait Pattern. 2024 10th IEEE RAS/EMBS International Conference for Biomedical Robotics and Biomechatronics (BioRob), 1063–1068. 10.1109/BioRob60516.2024.10719727

[25] Lewek M. D., Feasel J., Wentz E., Brooks F. P., & Whitton M. C. (2012). Use of Visual and Proprioceptive Feedback to Improve Gait Speed and Spatiotemporal Symmetry Following Chronic Stroke: A Case Series. Physical Therapy, 92(5), 748–756. 10.2522/ptj.20110206 22228605 PMC3345339

[26] Lewek M. D., Raiti C., & Doty A. (2018). The Presence of a Paretic Propulsion Reserve During Gait in Individuals Following Stroke. Neurorehabilitation and Neural Repair, 32(12), 1011–1019. 10.1177/1545968318809920 30558525 PMC6300055

[27] Lewek M. D., & Sawicki G. S. (2019). Trailing limb angle is a surrogate for propulsive limb forces during walking post-stroke. Clinical Biomechanics, 67, 115–118. 10.1016/j.clinbiomech.2019.05.011 31102839 PMC6635006

[28] McCain E. M., Dick T. J. M., Giest T. N., Nuckols R. W., Lewek M. D., Saul K. R., & Sawicki G. S. (2019). Mechanics and energetics of post-stroke walking aided by a powered ankle exoskeleton with speed-adaptive myoelectric control. Journal of NeuroEngineering and Rehabilitation, 16(1), 57. 10.1186/s12984-019-0523-y 31092269 PMC6521500

[29] Michelini A., Sivasambu H., & Andrysek J. (2022). THE SHORT-TERM EFFECTS OF RHYTHMIC VIBROTACTILE AND AUDITORY BIOFEEDBACK ON THE GAIT OF INDIVIDUALS AFTER WEIGHT-INDUCED ASYMMETRY. CANADIAN PROSTHETICS & ORTHOTICS JOURNAL, 5(1). 10.33137/cpoj.v5i1.36223 PMC10443516 37614474

[30] Mukherjee M., Eikema D. J. A., Chien J. H., Myers S. A., Scott-Pandorf M., Bloomberg J. J., & Stergiou N. (2015). Plantar tactile perturbations enhance transfer of split-belt locomotor adaptation. Experimental Brain Research, 233(10), 3005–3012. 10.1007/s00221-015-4370-1 26169104 PMC4575864

[31] Olney S. J., & Richards C. (1996). Hemiparetic gait following stroke. Part I: Characteristics. Gait & Posture, 4(2), 136–148. 10.1016/0966-6362(96)01063-6

[32] Padmanabhan P., Rao K. S., Gulhar S., Cherry-Allen K. M., Leech K. A., & Roemmich R. T. (2020). Persons post-stroke improve step length symmetry by walking asymmetrically. Journal of NeuroEngineering and Rehabilitation, 17(1), 105. 10.1186/s12984-020-00732-z 32746886 PMC7397591

[33] Park S., Liu C., Sánchez N., Tilson J. K., Mulroy S. J., & Finley J. M. (2021). Using Biofeedback to Reduce Step Length Asymmetry Impairs Dynamic Balance in People Poststroke. Neurorehabilitation and Neural Repair, 35(8), 738–749. 10.1177/15459683211019346 34060926 PMC8349786

[34] Patterson K. K., Gage W. H., Brooks D., Black S. E., & McIlroy W. E. (2010). Evaluation of gait symmetry after stroke: A comparison of current methods and recommendations for standardization. Gait & Posture, 31(2), 241–246. 10.1016/j.gaitpost.2009.10.014 19932621

[35] Patterson K. K., Parafianowicz I., Danells C. J., Closson V., Verrier M. C., Staines W. R., Black S. E., & McIlroy W. E. (2008). Gait Asymmetry in Community-Ambulating Stroke Survivors. Archives of Physical Medicine and Rehabilitation, 89(2), 304–310. 10.1016/j.apmr.2007.08.142 18226655

[36] Reisman D. S., Wityk R., Silver K., & Bastian A. J. (2007). Locomotor adaptation on a split-belt treadmill can improve walking symmetry post-stroke. Brain, 130(7), 1861–1872. 10.1093/brain/awm035 17405765 PMC2977955

[37] Sado T., Nielsen J., Glaister B., Takahashi K. Z., Malcolm P., & Mukherjee M. (2022). A passive exoskeleton can assist split-belt adaptation. Experimental Brain Research, 240(4), 1159–1176. 10.1007/s00221-022-06314-w 35165776 PMC9103932

[38] Santucci V., Alam Z., Liu J., Spencer J., Faust A., Cobb A., Konantz J., Eicholtz S., Wolf S., & Kesar T. M. (2023). Immediate improvements in post-stroke gait biomechanics are induced with both real-time limb position and propulsive force biofeedback. Journal of NeuroEngineering and Rehabilitation, 20(1), 37. 10.1186/s12984-023-01154-3 37004111 PMC10064559

[39] Schenck C., & Kesar T. M. (2017). Effects of unilateral real-time biofeedback on propulsive forces during gait. Journal of NeuroEngineering and Rehabilitation, 14(1), 52. 10.1186/s12984-017-0252-z 28583196 PMC5460355

[40] Skvortsov D. V., Kaurkin S. N., & Ivanova G. E. (2021). A Study of Biofeedback Gait Training in Cerebral Stroke Patients in the Early Recovery Phase with Stance Phase as Target Parameter. Sensors, 21(21), 7217. 10.3390/s21217217 34770524 PMC8588439

[41] Stimpson K. H., Heitkamp L. N., Embry A. E., & Dean J. C. (2019). Post-stroke deficits in the step-by-step control of paretic step width. Gait & Posture, 70, 136–140. 10.1016/j.gaitpost.2019.03.003 30856525 PMC6474800

[42] Woollacott M., & Tang P.-F. (1997). Balance control during walking in the older adult: Research and Its Implications. Physical Therapy, 77(6), 646–660. 10.1093/ptj/77.6.646 9184689

[43] Zeni J. A., Richards J. G., & Higginson J. S. (2008). Two simple methods for determining gait events during treadmill and overground walking using kinematic data. Gait & Posture, 27(4), 710–714. 10.1016/j.gaitpost.2007.07.007 17723303 PMC2384115

